# In-Field Calibration of Triaxial Accelerometer Based on Beetle Swarm Antenna Search Algorithm

**DOI:** 10.3390/s20030947

**Published:** 2020-02-10

**Authors:** Pengfei Wang, Yanbin Gao, Menghao Wu, Fan Zhang, Guangchun Li

**Affiliations:** Collage of Automation, Harbin Engineering University, Harbin 150001, China; hbforwpf@hrbeu.edu.cn (P.W.); wumenghao@hrbeu.edu.cn (M.W.); zhangfan41@hrbeu.edu.cn (F.Z.); lgc_67@hrbeu.edu.cn (G.L.)

**Keywords:** calibration, inertial measurement unit, triaxial accelerometer, intelligent optimization algorithm, beetle swarm antenna search algorithm

## Abstract

Traditional calibration method is usually performed with expensive equipments such as three-axis turntable in a laboratory environment. However in practice, in order to ensure the accuracy and stability of the inertial navigation system (INS), it is usually necessary to recalibrate the inertial measurement unit (IMU) without external equipment in the field. In this paper, a new in-field recalibration method for triaxial accelerometer based on beetle swarm antenna search (BSAS) algorithm is proposed. Firstly, as a new intelligent optimization algorithm, BSAS algorithm and its improvements based on basic beetle antennae search (BAS) algorithm are introduced in detail. Secondly, the nonlinear mathematical model of triaxial accelerometer is established for higher calibration accuracy, and then 24 optimal measurement positions are designed by theoretical analysis. In addition, the calibration procedures are improved according to the characteristics of BSAS algorithm, then 15 calibration parameters in the nonlinear method are optimized by BSAS algorithm. Besides, the results of BSAS algorithm and basic BAS algorithm are compared by simulation, which shows the priority of BSAS algorithm in calibration field. Finally, two experiments demonstrate that the proposed method can achieve high precision in-field calibration without any external equipment, and meet the accuracy requirements of the INS.

## 1. Introduction

Inertial navigation is a very common method of navigation. Different from wireless navigation system [1], inertial navigation system (INS) obtains speed and direction information through integration, so inertial navigation is an autonomous navigation method. Due to the characteristics of inertial navigation, inertial navigation is widely used in various fields [2,3,4].

Triaxial accelerometer is one of the important components of the inertial measurement unit (IMU). The triaxial accelerometer can be used to judge the motion state of people or objects by measuring specific force information, and its accuracy will directly affect the performance of INS [5]. Because the calibration parameters of triaxial accelerometer are not invariable, some studies show that the calibration parameters such as scale factors and biases of the sensor will change in different environments [6,7]. Therefore, it is usually necessary to recalibrate the triaxial accelerometer if the calibration parameters change greatly over time.

Calibration with external equipment is the most common and traditional method. In the laboratory environment, the IMU can be calibrated by using three-axis turntable to conduct position experiment and speed experiment [8,9], and the calibration accuracy is affected by the accuracy of three-axis turntable. Because the traditional method is limited by the dependence on the equipment, more and more researchers have paid close attention to calibration methods that do not need any external equipment. In 1995, Ferraris et al. estimated biases and scale factors of IMU by placing the IMU in six different positions [10]. Although the IMU mathematical model they used is too simple, they achieved calibration without turntable. In 2002, Shin et al. added the installation angle errors to the calibration mathematical model, and then proposed a in-field calibration method by making gravity acceleration and earth rotation velocity as a reference [11]. In this method, the earth gravity and rotation velocity measured at different positions are used to establish the observation equation, and then the equation is solved by the least square method. Syed et al. in 2007 employed Newton iterative optimization method to calibrate the nine parameters of IMU without any equipment, but the scheme is very complex, and the calibration accuracy is not very high [12]. In 2010, Won et al. put forward a new iterative method by using the calibration mathematical model of scale factors and biases. They successfully calibrate the triaxial accelerometer by measuring the specific force in six different angles, but this method is only useful for low cost IMU [13]. In 2013, Cai et al. presented an improved multi position calibration scheme and realized the calibration of the nonlinear scale factors. The scheme greatly improves the accuracy of the IMU, but the problem of complex calculation is still not solved [14]. In 2017, Ye et al. tried to linearize the mathematical model of triaxial accelerometer and proposed a six-orientation optimal experimental scheme. Although they finally achieved rapid automatic calibration through iteration, the scheme is only applicable to the IMU with low accuracy [15]. Different from the research of Ye et al., in 2019, Wang et al. developed a calibration method for the IMU with a nonlinear mathematical model. This method could get high calibration accuracy by Levenberg-Marquardt (LM) optimization algorithm, but the mathematical model which it used is too complicated [16]. To sum up, it can be found that the optimization algorithm, as a common method for processing data and optimizing indicators [17,18], is now widely used in sensor calibration.

In this paper, a new in-field calibration method based on beetle swarm antenna search (BSAS) algorithm is proposed. The rest of this paper is arranged as follows. In Section 2, beetle antennae search (BAS) algorithm as a new meta-heuristic intelligent optimization algorithm is introduced firstly. Then, BSAS algorithm and its advantages are described in detail. The in-field calibration procedures of triaxial accelerometer based on the proposed method will be presented in the Section 3. Next, in the Section 4, simulations based on the basic BAS and BSAS algorithm are carried out, and the performance of the two algorithms will be compared by analyzing the simulation results. After that, in Section 5, two experiment results are reported separately to prove the effectiveness of the proposed method. Finally, the conclusions of this paper are given in Section 6.

## 2. Related Works of BSAS Algorithm

### 2.1. Beetle Antennae Search Algorithm

Beetle antennae search algorithm is a new meta-heuristic intelligent optimization algorithm proposed by Jiang et al.  in 2017, it was inspired by beetle’s foraging behavior [19]. When the beetle is looking for food, the beetle judges the location of food based on the intensity of food smell received by its two antennae. If the smell received by the left antennae is stronger than that received by the right antennae, the beetle will choose to fly to the left, otherwise it will fly to the right. In practical application, the food can be expressed as a function to be optimized. By imitating the behavior of beetles, after constantly comparing the intensity of food smell (i.e. value of the function to be optimized), the optimal value of the function can be obtained finally. At the beginning of each iteration, the search direction of the beetle is randomly initialized and normalized, as shown in Equation (1).

(1) $$\vec{d_{ir}}=\frac{rnd\left(n,1\right)}{{∥rnd\left(n,1\right)∥}_{2}}$$

where *n* represents the dimension of the optimization problem, rnd(·) is a random function used to generate random directions. The positions of the left and right antennae can be expressed by Equations (2) and (3).

(2) $$x_{l}^{k}=x^{k}-d^{k}\cdot \vec{d_{ir}}$$

(3) $$x_{r}^{k}=x^{k}+d^{k}\cdot \vec{d_{ir}}$$

(4) $$\left\{\begin{array}{c} d^{k}=c_{d}d^{k-1}+d_{0}\;\\ c_{d}\in \left(0,1\right) \end{array}\right.$$

where $x_{l}$ and $x_{r}$ are the positions of the left and right antennae of the beetle, $x^{k}$ represents the position of the beetle in space. $d^{k}$ is the distance between the two antennae and the mass center of the beetle, which can also be called the search distance. *k* is the number of iterations, where $x_{r}^{k}$, $x_{l}^{k}$, $x^{k}$ and $d^{k}$ are changed with iteration. The search distance is updated according to Equation (4), where $d_{0}$ represents the minimum resolutions of search distance. $c_{d}$ is the attenuation coefficient of search distance, which needs to be set in advance.

According to the foraging behavior of beetles mentioned above, the position of beetles at the next moment can be denoted as Equation (5):

(5) $$x^{k}=x^{k-1}+s^{k}\cdot \vec{d_{ir}}\cdot sign\left(f\left(x_{r}^{k-1}\right)-f\left(x_{l}^{k-1}\right)\right)$$

(6) $$\left\{\begin{array}{c} s^{k}=c_{s}s^{k-1}+s_{0}\;\\ c_{s}\in \left(0,1\right) \end{array}\right.$$

where $s^{k}$ is the search step size of the kth iteration, its updating rule is shown as Equation (6), and $s_{0}$ is the minimum resolutions of step size. The attenuation coefficient $c_{s}$ of the search step size also needs to be set in advance. In Equation (5), sign represents symbolic function, and f(·) is the function which need to be optimized. If f(r) is greater than f(l), the next iteration will choose the direction close to f(r), otherwise it will choose the opposite direction. The specific steps of basic BAS algorithm are shown in Algorithm 1.

Zhang et al. theoretically analyzed the convergence of the basic BAS algorithm in reference [20], so it is not the focus of this paper.

Owing to simple principle, fast convergence speed and small amount of calculation, basic BAS has been applied to many fields since it was proposed. Wu et al.  used basic BAS algorithm to optimize the navigation path of unmanned aerial vehicles, and realized fast and efficient path planning [21]. Fei et al. applied basic BAS algorithm in the prediction of dissolved gas content in power transformer, and they demonstrate the superiority of basic BAS through experiments [22]. Wu et al. proposed to employ basic BAS algorithm to optimize the weights of neural network classifier, which improves training speed of traditional neural network [23]. In order to improve the robustness of PID (Proportion Integration Differentiation) controller, Fan et al. utilized basic BAS algorithm to optimize three parameters in PID control, and finally achieved the effective adjustment of the controller [24]. Li et al. employed basic BAS algorithm to optimize the proposed model in the energy scheduling problem, and energy costs are reduced through their proposed method [25]. Chen et al. invented a NLSSVM (Nonlinear Least Squares Support Vector Machine)-BAS algorithm, which can optimize the parameters in the radial basis function by using basic BAS algorithm [26]. This new nonlinear least squares support vector machine algorithm performes well in the parameter identification of ship model. Sun et al. used basic BAS algorithm to optimize the structure of back propagation neural network, and the BPNN (Back Propagation Neural Network)-BAS model which they proposed is more accurate than multiple regression and support vector machine models in practical application [27].

In the past few years, basic BAS algorithm has attracted great interest because of its unique advantages. However, compared with other swarm intelligence algorithms, the performance of basic BAS is not always the best. For instance, recent studies show that the results of basic BAS algorithm are usually inferior to particle swarm optimization (PSO) and genetic algorithm (GA) in terms of global optimization [28]. It means that for optimizing complex problems, basic BAS algorithm is easily trapped in local optimal value. In order to overcome the shortcomings of the basic BAS algorithm, a new BSAS algorithm is proposed.
**Algorithm 1** BAS algorithm**Input:** Define cost function f(·). Set the dimension of parameters to be optimized as *n*.**Output:** Optimal solution $x_{best}$ and $f_{best}$.1:**Initialize:**Initialize beetle position $x^{0}$;Initialize search step size $s^{0}$ and its attenuation coefficient $c_{s}$;Initialize search distance $d^{0}$ and its attenuation coefficient $c_{d}$;Initialize the maximum number of iterations $k_{max}$;Initialize optimum results for $x_{best}$ and $f_{best}$.2:**if**$k<=k_{max}$**then**3:   Randomly generate the search direction ${\vec{d}}_{ir}$4:   Calculate the position of the two antennae $x_{l}^{k}$ and $x_{r}^{k}$ respectively by Equation (2) and (3).5:   Calculate the beetle position $x^{k}$ for this iteration by Equation (5), and calculatethe function value $f(x^{k})$.6:   Compare $f(x^{k})$ and $f_{best}$.7:   **if** $f(x^{k})$ is closer to the ideal result than $f_{best}$ **then**8:   $x_{best}$ =$x^{k}$9:   $f_{best}$ =$f(x^{k})$10:   **end if**11:   Update search step size $s^{k}$ by Equation (6).12:   Update search distance $d^{k}$ by Equation (4).13:   k=k+114:**end if**

### 2.2. Beetle Swarm Antenna Search Algorithm

BSAS algorithm was proposed by Wang et al. in 2018 [28,29]. It is an improved algorithm based on the basic BAS algorithm. By introducing the idea of swarm intelligence and a improved update strategy of step size, the performance of BSAS algorithm is greatly improved compared to the basic BAS algorithm in solving complex problems [28,30,31].

In order to to improve the ability of optimization, BSAS algorithm expands a single beetle in basic BAS algorithm to beetle group:

(7) $$\left\{\begin{array}{c} x_{ri}^{k}=x_{i}^{k}+d^{k}{\vec{d}}_{iri}\;\\ x_{li}^{k}=x_{i}^{k}-d^{k}{\vec{d}}_{iri}\;\\ x_{i}^{k}=x_{i}^{k-1}+s^{k}\cdot {\vec{d}}_{iri}\cdot \mathrm{sign}\left(f\left(x_{ri}^{k-1}\right)-f\left(x_{li}^{k-1}\right)\right) \end{array}\right.$$

Different from Equation (2) and (3) and (5), *i* in Equation (7) represents the number of beetles, i∈1,2,3,⋯N. By increasing the number of beetles, all beetles in each iteration use the same search method as basic BAS algorithm. Compared with a single beetle, a large number of beetles can compete internally, then more suitable beetles are selected.

Furthermore, for the sake of avoiding the local optimal value, three new parameters for step size update strategy are introduced into BSAS algorithm:

Parameter 1: $p_{pos}$ is a probability constant. According to previous experience, in each iteration, the beetle with the best position in the beetle group will be selected as the starting position of the next iteration. However, the actual situation demonstrates that because of the interference of local optimal value, choosing the optimal value in each iteration does not mean that the global optimal value will be obtained in the end. Therefore, for each iteration, if *M* beetles find a better position, a random number *a* will be generated between 0 and 1, and then it should be compared with $p_{pos}$. If *a* is smaller than $p_{pos}$, the beetle which has the best position will be selected as the starting position of the next iteration, otherwise, one of the *M* beetles will be randomly selected.

Parameter 2: $p_{st}$ is also a probability constant, which is used to control the update of search step size. At a certain iteration, if all beetles do not find a better position, it will be considered that the current search step size is too large. Thus the position will be not updated in next iteration, but the step size will be reduced and then optimal value continues to be searched. However, the search step size is unconditionally updated in each iteration (which means the search step size is constantly reduced) may result in missing the global optimal value. Therefore, similar to $p_{pos}$, the update of search step size can be controlled by setting $p_{st}$. When all beetles can’t find a better position, a random number *b* which in (0,1) will be generated. If *b* is smaller than $p_{st}$, the search step size wlii be updated, otherwise, it will be kept for the next iteration.

Parameter 3: Parameter 2 $p_{st}$ can improve the global optimization ability of the algorithm to a certain extent, but the efficiency of algorithm will be reduced by setting a inappropriate $p_{st}$. Consequently, parameter $n_{st}$ is presented to ensure the normal operation of the algorithm. When the number of invalid searches by using the same step size exceeds $n_{st}$, the search step size will be forced to update in next iteration.

The detailed steps of BSAS algorithm are shown as Algorithm 2.

Through the above introduction, it can be find that the advantages of the BSAS algorithm compared with the basic BAS algorithm are mainly reflected in two aspects. Firstly, by increasing the number of beetles, the optimization ability of the algorithm is greatly improved. Secondly, three new parameters added to the step size updating strategy can make the algorithm avoid local optimization effectively. Therefore, when dealing with complex problems such as the calibration of three axis accelerometers, the BSAS algorithm has obvious superiority.
**Algorithm 2** BSAS algorithm**Input:** Define cost function f(·). Set the dimension of parameters to be optimized as *n*.**Output:** Optimal solution $x_{best}$ and $f_{best}$.1:**Initialize:**Initialize the position of all beetles $x_{i}^{0}$ and $p_{pos}$;Initialize search step size $s^{0}$ and its attenuation coefficient $c_{s}$;Initialize $p_{st}$ and $n_{st}$;Initialize search distance $d^{0}$ and its attenuation coefficient $c_{d}$;Initialize the maximum number of iterations $k_{max}$;Initialize optimum results for $x_{best}$ and $f_{best}$.2:**if**$k<=k_{max}$**then**3:   Randomly generate the search direction ${\vec{d}}_{iri}$4:   Calculate the position of the two antennae of all beetles, $x_{li}^{k}$ and $x_{ri}^{k}$ respectively by Equation (7).5:   Calculate the position of all beetles $x_{i}^{k}$ for this iteration by Equation (7), and calculate the function value $f(x_{i}^{k})$.6:   Compare current all $f(x_{i}^{k})$ and $f_{best}$.7:   **if** ∃$f\left(x_{i}^{k}\right)<f_{best}$ **then**8:   **if** $rand\left(1\right)=a<p_{pos}$ **then**9:     $x_{best}$ =$argmin(f\left(x_{i}^{k}\right))$10:    $f_{best}$ =$min(f\left(x_{i}^{k}\right))$11:   **else**12:    $x_{best}$ =$sample(x_{i}^{k})$13:    $f_{best}$ =$\left(f\left(x_{sample}^{k}\right)\right)$14:   **end if**15:  **else**16:   **if** $rand\left(1\right)=b<p_{st}\left|\right|i>n_{st}$ **then**17:    Update search step $s^{k}$ by Equation (6).18:    Update search distance $d^{k}$ by Equation (4).19:   **else**20:    i=i+121:   **end if**22:    **end if**23:    k=k+124:**end if**

## 3. Methodology

This section will first introduce the mathematical calibration model of the triaxial accelerometer used in this paper, and then cost function is presented according to it. Next, 24 optimal measurement positions are designed by theoretical analysis. Finally, the in-field calibration steps of the triaxial accelerometer based on the BSAS algorithm are given.

### 3.1. Calibration Model of Triaxial Accelerometer

In essence, IMU calibration technology is the process of optimizing the calibration parameters in the calibration model, so the selection of the mathematical model will directly affect calibration result. In order to improve the calibration accuracy, the second-order nonlinear scale factor [16] is introduced into the calibration model in this paper:

(8) $$N_{a}=K_{a}\left(T_{a}A+b_{0}\right)+K_{2a}A^{2}+\sigma_{a}$$

where $N_{a}$ is the measurement output value of the accelerometer, $K_{a}$ is the scale factor, *A* is the measuring specific force input value, $b_{0}$ is the bias, $K_{2a}$ is the second-order nonlinear scale factor, and $\sigma_{a}$ is random noise. Besides, $T_{a}$ is the installation angle error of the triaxial accelerometer [14], it is denoted as follows:

(9) $$T_{a}=\left[\begin{array}{ccc} 1 & Saxz & Saxy\\ Sayz & 1 & Sayx\\ Sazy & Sazx & 1 \end{array}\right]$$

Then by combining Equation (8) and Equation (9), and ignoring the random noise $\sigma_{a}$, the nonlinear calibration model of triaxial accelerometer can be obtained as follows:

(10) $$\left[\begin{array}{c} N_{ax}\\ N_{ay}\\ N_{az} \end{array}\right]=\left[\begin{array}{ccc} K_{ax} & S_{axz}K_{ax} & S_{axy}K_{ax}\\ S_{ayz}K_{ay} & K_{ay} & S_{ayx}K_{ay}\\ S_{azy}K_{az} & S_{azx}K_{az} & K_{az} \end{array}\right]\left[\begin{array}{c} A_{x}\\ A_{y}\\ A_{z} \end{array}\right]+\left[\begin{array}{c} b_{x0}K_{ax}\\ b_{y0}K_{ay}\\ b_{z0}K_{az} \end{array}\right]+\left[\begin{array}{c} K_{2ax}A_{x}^{2}\\ K_{2ay}A_{y}^{2}\\ K_{2az}A_{z}^{2} \end{array}\right]$$

### 3.2. Cost Function

According to the measurement principle of accelerometer [32], theoretically, in the static case, the relationship between the calculated value of specific force and the local gravity acceleration *g* should be as follows:

(11) $$A_{x}^{2}+A_{y}^{2}+A_{z}^{2}=g^{2}$$

However, due to the measurement error and calculation error, there is an error term $e_{i}$ between the gravity acceleration and the specific force that calculated from the calibration model, as shown in Equation (12).

(12) $$e_{i}=\sqrt{A_{xi}^{2}+A_{yi}^{2}+A_{zi}^{2}}-g$$

From the Equation (10), $e_{i}$ contains 15 calibration parameters. Therefore, the in-field calibration method proposed in this paper is to use BSAS algorithm optimizing 15 calibration parameters, and the cost function is as follows:

(13) $$f=\min\sum_{i=1}^{n}∥e_{i}∥$$

where *i* represents the different measuring positions.

### 3.3. Scheme of Optimal Measuring Position

In order to get more accurate calibration parameters for the in-field calibration, in theory, the measurement position of IMU should cover the whole 3-dimensional space. But it’s actually impossible, because the number of consecutive measurement positions is infinite. Consequently, for the sake of reducing the cost of calibration without affecting the calibration results, many researchers use multi-position calibration scheme to carry out in-field calibration [33,34,35]. The choice of measurement position will affect the final result of calibration, so this paper designs the optimal measurement position scheme through the following rules: Firstly, because the cost function Equation (13) contains 15 calibration parameters to be optimized, the number of measurement positions cannot be less than 15 to avoid calculation singularity. Secondly, the calibration mathematical model Equation (10) ignores the coupling error, so the measurement position in the symmetrical direction should be selected as much as possible to reduce the influence of the coupling error on the calibration accuracy. Thirdly, in the static case, the sensitivity of the 15 calibration parameters to the local gravity acceleration are different, the analysis is as follows:

(14) $$\left\{\begin{array}{c} \;A_{x}=\frac{\left(N_{ax}-S_{axz}K_{ax}A_{y}-S_{axy}K_{ax}A_{z}-b_{x0}K_{ax}-K_{2ax}A_{x}^{2}\right)}{K_{ax}}\;\\ \;A_{y}=\frac{\left(N_{ay}-S_{ayz}K_{ay}A_{x}-S_{ayx}K_{ay}A_{z}-b_{y0}K_{ay}-K_{2ay}A_{y}^{2}\right)}{K_{ay}}\;\\ \;A_{z}=\frac{\left(N_{az}-S_{azy}K_{az}A_{x}-S_{azx}K_{ay}A_{y}-b_{z0}K_{az}-K_{2az}A_{z}^{2}\right)}{K_{az}} \end{array}\right.$$

Equation (14) is derived from Equation (10), and it represents the calculated specific force value of triaxial accelerometer. The relationship between the acceleration of gravity and it can be expressed as a function $F_{A}$.

(15) $$F_{A}=A_{x}^{2}+A_{y}^{2}+A_{z}^{2}-g^{2}$$

Equation (16) can be obtained by substituting Equation (14) into equation Equation (15).

(16) $$\begin{array}{cc} F_{A}= & {\left[\frac{\left(N_{ax}-S_{axz}K_{ax}A_{y}-S_{axy}K_{ax}A_{z}-b_{x0}K_{ax}-K_{2ax}A_{x}^{2}\right)}{K_{ax}}\right]}^{2}\\ & +{\left[\frac{\left(N_{ay}-S_{ayz}K_{ay}A_{x}-S_{ayx}K_{ay}A_{z}-b_{y0}K_{ay}-K_{2ay}A_{y}^{2}\right)}{K_{ay}}\right]}^{2}\\ & +{\left[\frac{\left(N_{az}-S_{azy}K_{az}A_{x}-S_{azx}K_{az}A_{y}-b_{z0}K_{az}-K_{2az}A_{z}^{2}\right)}{K_{az}}\right]}^{2}-g^{2} \end{array}$$

Next, the partial derivative of $K_{ax}$ is calculated, as shown in Equation (17). It is worth noting that the smaller terms, such as installation error angle, are ignored.

(17) $$\begin{array}{cc} \frac{\partial F_{A}}{\partial K_{ax}}= & {\frac{-2\left(N_{ax}-S_{axz}K_{ax}A_{y}-S_{axy}K_{ax}A_{z}-b_{x0}K_{ax}-K_{2ax}A_{x}^{2}\right)}{K_{ax}^{3}}}^{2}\\ \;\; & +\frac{2\left(N_{ax}-S_{axz}K_{ax}A_{x}-S_{axy}K_{ax}A_{z}-b_{x0}K_{ax}-K_{2ax}A_{x}^{2}\right)\left(-b_{x0}\right)}{K_{ax}^{2}}\\ \;\; & \approx \frac{-2\left(A_{x}^{2}+A_{x}b_{x0}\right)}{K_{ax}} \end{array}$$

Similarly, the calculation results for the other 14 calibration parameters are as follows:

(18) $$\frac{\partial F_{A}}{\partial K_{ay}}\approx \frac{-2\left(A_{y}^{2}+A_{y}b_{y0}\right)}{K_{ay}};\frac{\partial F_{A}}{\partial K_{az}}\approx \frac{-2\left(A_{z}^{2}+A_{z}b_{z0}\right)}{K_{az}}$$

(19) $$\frac{\partial F_{A}}{\partial b_{x0}}\approx -2A_{x};\frac{\partial F_{A}}{\partial b_{y0}}\approx -2A_{y};\frac{\partial F_{A}}{\partial b_{z0}}\approx -2A_{z}$$

(20) $$\begin{array}{cc} \; & \frac{\partial F_{A}}{\partial S_{axz}}\approx -2A_{x}A_{y};\frac{\partial F_{A}}{\partial S_{axy}}\approx -2A_{x}A_{z};\frac{\partial F_{A}}{\partial S_{ayz}}\approx -2A_{y}A_{x}\\ \; & \frac{\partial F_{A}}{\partial S_{ayx}}\approx -2A_{y}A_{z};\frac{\partial F_{A}}{\partial S_{azy}}\approx -2A_{z}A_{x};\frac{\partial F_{A}}{\partial S_{azx}}\approx -2A_{z}A_{y} \end{array}$$

(21) $$\frac{\partial F_{A}}{\partial K_{2ax}}\approx \frac{-2A_{x}^{3}}{K_{ax}};\frac{\partial F_{A}}{\partial K_{2ay}}\approx \frac{-2A_{y}^{3}}{K_{ay}};\frac{\partial F_{A}}{\partial K_{2az}}\approx \frac{-2A_{z}^{3}}{K_{az}}$$

Equations (17)–(21) indicates that when the three axes of the triaxial accelerometer are parallel to the direction of gravity acceleration, the sensitivities of the scale factors, biases and second-order nonlinear scale factors are maximized. When the gravity acceleration is parallel to the plane formed by two axes of the triaxial accelerometer, and the angle between the two axes and gravity acceleration is 45 degrees, the installation angle errors have the highest sensitivity.

**Figure 1:**
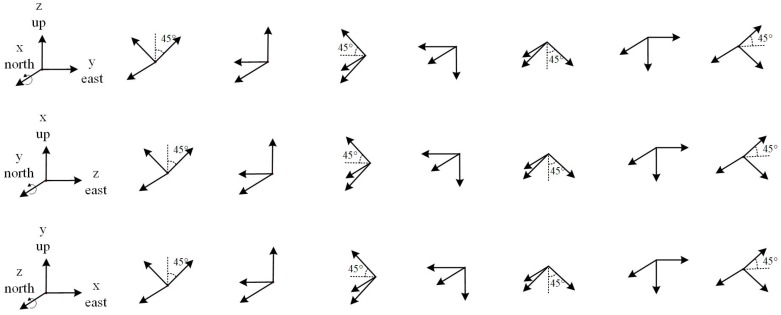
Scheme of measurement position.

Based on the above analysis, 24 measurement positions are designed as shown in Figure 1. To start with, the 24 positions of the triaxial accelerometer are divided into three groups, and the three axes x,y,z are respectively used as the rotation axes of the three groups. Next, the rotation axes of the three groups all point north, then the triaxial accelerometers rotate 45 degrees anticlockwise successively around the rotation axis, each group rotates 7 times, and the three groups have 24 positions in total. In addition, since the proposed method of in-field calibration does not rely on external equipment, the direction of each position does not need to be precisely aligned.

### 3.4. Calibration Procedures Based on BSAS Algorithm

Due to the mathematical model used in this paper contains 15 calibration parameters, considering the complexity and particularity of calibration, it is necessary to analyze and improve the calibration procedure according to the characteristics of BSAS algorithm. The complexity and particularity of the problem are reflected in the following two aspects:

1. Because of the unique step size update strategy in BSAS algorithm, it requires that different parameters which need to be optimized should have similar magnitude, otherwise the optimization results will be affected.
2. Relevant research shows that different calibration parameters have different degrees of influence on the cost function, which means that different calibration parameters have different effects on calibration accuracy [5].

In view of the above two points, the following improvements can be made by combining the characteristics of the BSAS algorithm: First of all, in light of the sequence of magnitude from large to small, 15 calibration parameters are divided into four categories: the scale factors, the biases, the second-order nonlinear scale factors and the installation angle errors. Next, a reasonable optimization sequence is designed according to the influence degree of parameters on the calibration accuracy: the scaling factors are optimized first, the biases are behind the scaling factors, next are the installation angle errors, and the second-order nonlinear scale factors are the last.

To sum up, the calibration steps based on the BSAS algorithm are as follows:

Step 1: The optimization of scale factors is implemented by using BSAS algorithm. In order to avoid the influence of the other three types of calibration parameters on the results, the biases, the installation angle errors, and the second-order nonlinear scale factors need to be initialized in advance.
Step 2: The biases are optimized based on the BSAS algorithm. In the initialization phase, the installation angle errors and the second-order nonlinear scale factors are preseted, while the scale factors are set to the optimization result in Step 1.
Step 3: First, the optimization results of the scale factors and the biases are used as preset values, and then the installation angle errors are optimized after initializing the second-order nonlinear scale factors.
Step 4: Optimization of the second-order nonlinear scale factors is performed by BSAS algorithm, and the optimization results of the first three types of calibration parameters will be loaded in advance in the initialization stage.

**Figure 2:**
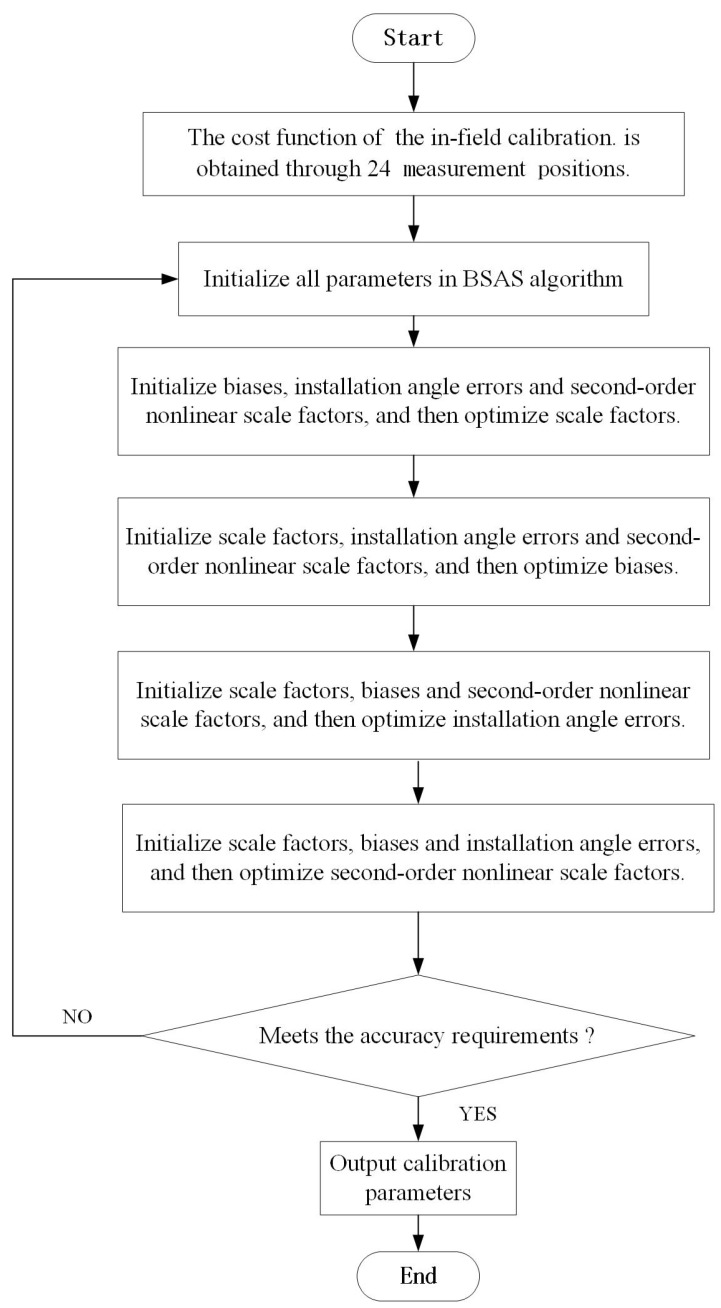
Flowchart of the proposed method.

It is worth noting that for the purpose of getting higher calibration accuracy, 15 parameters may need to be optimized many times. Therefore, the above steps can be repeated until the requirements are met. The 15 parameters optimization flowchart of the in-field calibration based on BSAS algorithm is shown in Figure 2.

## 4. Simulation and Analysis

In this section, the effectiveness of the proposed method is verified by simulation. The simulation environment is explained first, including parameter initialization and details of simulation. After that, the simulation results of BSAS algorithm and basic BAS algorithm are compared and analyzed.

### 4.1. Simulation Environment

In simulation, the real values of 15 calibration parameters are set as shown in Table 1, and the random bias of accelerometer is set as 10 ug.

**Figure 3:**
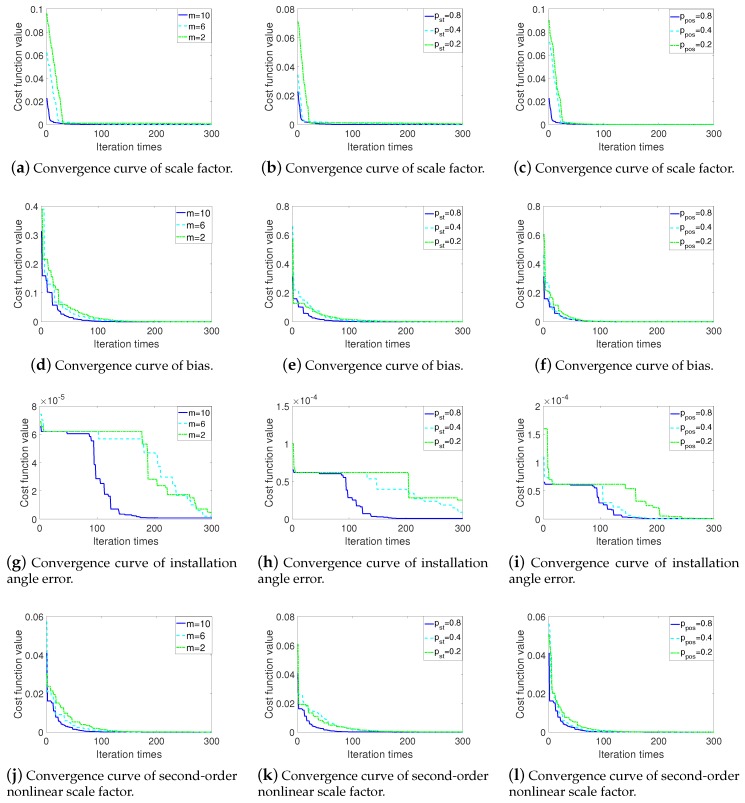
Convergence curve of BSAS with different parameters.

Figure 3 shows the optimization effect of BSAS algorithm with different parameters. In terms of position update probability constant $p_{pos}$, as shown in Figure 3c,f,i,l, $p_{pos}$ is directly affect the convergence speed and efficiency of the algorithm. With the increase of $p_{pos}$, the convergence speed of the algorithm has significantly improved. In addition to the convergence speed, the number of beetles *m* and step size update probability constant $p_{st}$ also affect the optimization ability of the algorithm. As shown in Figure 3a,d,g,j, when the number of beetles *m* is 10, after 300 iterations, the cost function values for optimizing the four types of calibration parameters are $8.365\times 10^{-7}$, $7.564\times 10^{-7}$, $8.112\times 10^{-7}$ and $7.344\times 10^{-7}$. When *m* is 6, the values are $3.262\times 10^{-6}$, $1.545\times 10^{-6}$, $2.036\times 10^{-6}$ and $1.134\times 10^{-6}$, and when m=2, the values are $6.112\times 10^{-4}$, $6.335\times 10^{-6}$, $4.513\times 10^{-6}$, and $1.620\times 10^{-6}$. Similar to the number of beetles, Figure 3b,e,h,k shows the parameter $p_{st}$ also affects the optimization results.

According to the above simulation results analysis, the parameters in BSAS algorithm are set as shown in Table 2. In addition, the simulation in this paper is completed by using MATLAB software, so the minimum resolution $s_{0}$ and $d_{0}$ are set to $2.22\times 10^{-16}$.

It is worth remarking 15 calibration parameters are divided into 4 categories and optimized in turn according to the description of Section 3.4. In order to avoid the interaction between different types of parameters in the optimization process, the calibration parameters of the accelerometer should be preseted in the initialization phase. The application background of the proposed scheme is to recalibrate the triaxial accelerometer without any equipment in the field, so the calibration parameters provided by the manufacturer (which are obtained by using three-axis turntable) are considered as the preset values.

### 4.2. Simulation Results and Analysis

Because the search path of meta-heuristic intelligent optimization algorithm is random every iteration [36], in order to verify the stability and robustness of the proposed scheme, 50 simulation experiments are performed.

**Figure 4:**
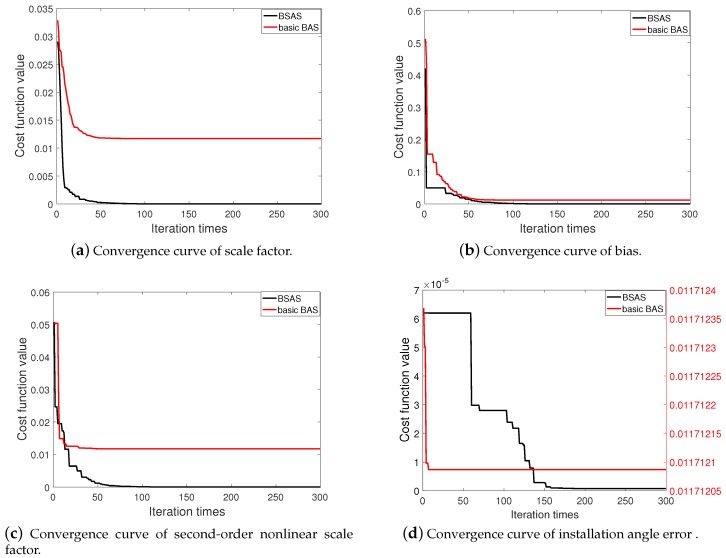
Convergence curve of BSAS and BAS algorithm.

For simplicity, Figure 4 is the convergence curve of the four types of calibration parameters in a simulation experiment. By comparison, it can be found that the curves of two different colors both have the fastest descent speed in the first 20 iterations, and the convergence speed of the BSAS algorithm exceeds the basic BAS algorithm. After 20 iterations, the basic BAS algorithm completes the optimization in advance and is trapped in the local optimal value. In Figure 4d, for the calibration of installation angle error parameters with high accuracy requirements, the basic BAS algorithm can hardly optimize it, while BSAS algorithm can jump out of the local optimal value and continue to search after 60 iterations. Therefore, in terms of convergence speed and global optimization capability, the BSAS algorithm is superior than that of basic BAS algorithm.

**Figure 5:**
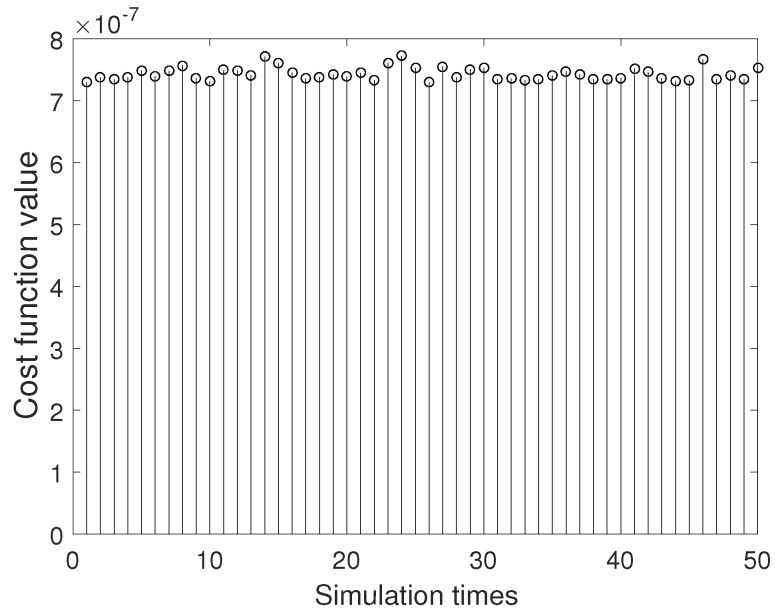
50 simulation results of cost function for BSAS algorithm.

**Figure 6:**
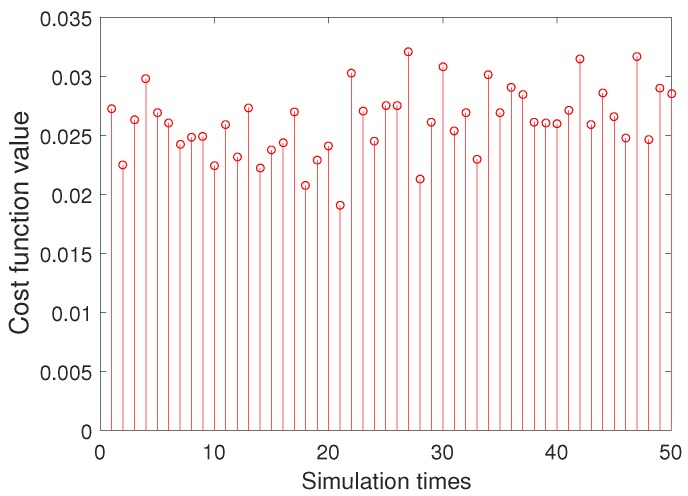
50 simulation results of cost function for basic BAS algorithm.

**Figure 7:**
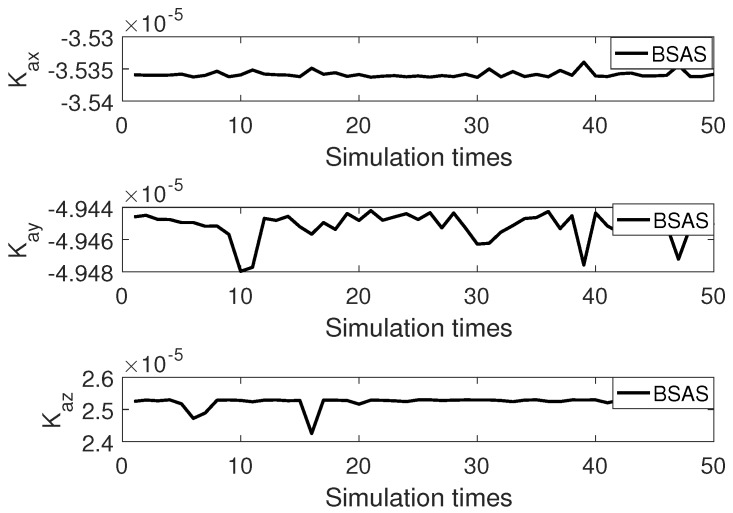
50 times simulation error curve of scale factors.

Figure 5 and Figure 6 are 50 simulation results of cost function for BSAS algorithm and basic BAS algorithm. As can be seen from the two figures, the 50 cost function results of the BSAS algorithm are varied from $7.2\times 10^{-7}$ to $7.4\times 10^{-7}$, and the results of the basic BAS algorithm range between $2\times 10^{-2}$ and $3.5\times 10^{-2}$. Therefore, the stability and robustness of the BSAS algorithm is far better than the basic BAS algorithm from the discrete degree of the results. The error curve between 50 simulation results based on BSAS algorithm and real value for three scale factors is shown in Figure 7.

The detailed results of 50 simulation are given in Table 3. First of all, the root mean square error (RMSE) indicates that the in-field calibration scheme based on the BSAS algorithm can accurately calibrate 15 calibration parameters including the scale factors, biases, installation angle errors, and second-order nonlinear scale factors. Where the accuracy of scale factors is $10^{-5}$ in magnitude, the accuracy magnitude of biases can reach $10^{-7}∼10^{-8}$, the accuracy magnitude of the installation angle errors is in the range of $10^{-5}∼10^{-6}$, and the accuracy magnitude of second-order nonlinear scale factors is distributed in $10^{-6}∼10^{-7}$. However, the calibration results of triaxial accelerometer by using the basic BAS algorithm can not achieve the high precision calibration. Especially, when optimizing precise parameters like the installation angle errors, the basic BAS algorithm usually fails to converge. Furthermore, the robustness and stability of the algorithm are illustrated by the standard deviation (SD). The standard deviation magnitude of 15 calibration parameters by the BSAS algorithm is in the range of $10^{-5}∼10^{-9}$, which is far smaller than the result of the basic BAS algorithm.

By comparing the simulation results of the two algorithms, the superiority of BSAS algorithm in solving complex problems is proved. On the one hand, the BSAS algorithm improves the stability by increasing the number of beetles, but it should be noted that more beetles will increase the calculation amount [28]. On the other hand, the new step size update strategy makes the BSAS algorithm have better global optimization ability.

Through the above analysis, it can be concluded that the BSAS algorithm can well meet the requirements of in-field calibration proposed in this paper, while the BSAS algorithm can not.

## 5. Experimental Analysis

**Figure 8:**
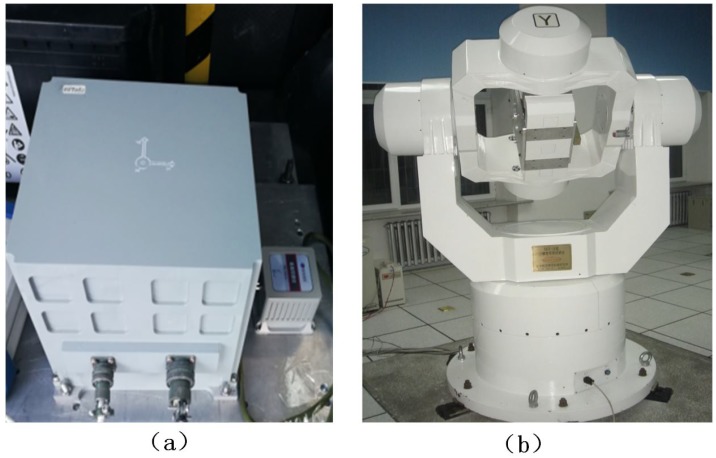
Experimental equipment. (**a**) Self-developed SINS. (**b**) Three-axis turntable.

In this section, 36 position triaxial acceleration measurement experiment and static navigation experiment based on strapdown inertial navigation system (SINS) were performed separately to prove the feasibility of the proposed scheme. In all experiments, the SINS based on triaxial accelerometer is independently developed by Harbin Engineering University, and the main performance indexes of the gyroscope and accelerometer are shown in Table 4. Furthermore, both experiments were implemented on a three-axis turntable in laboratory, as shown in Figure 8, and the indoor temperature was kept at 24 degrees centigrade.

### 5.1. 36 Position Measurement Experiment

In the 36 position triaxial accelerometer measurement experiment, the triaxial accelerometer is placed on a three-axis turntable firstly, and then the measurement data of triaxial accelerometer is collected at 36 different positions.

It should be pointed out that the determination rule of 36 positions is roughly the same as that of 24 positions in Section 3.3, the difference is that the rotation angle around the rotation axis in turn is 30 degrees. There are 12 positions in each group, and three groups have 36 positions in total.

(22) $$error=\|(\sqrt{A_{x}^{2}+A_{y}^{2}+A_{z}^{2}}-g)\|$$

According to Equations (11)–(12), in the static case, the absolute value of the error between the calculated specific force value $A_{x}$, $A_{y}$, $A_{z}$ and the local gravity acceleration *g* can be obtained by Equation (22).

The 36 measurement positions are divided into three groups according to the rotation axis, and the error results are shown as Figure 8.

**Figure 9:**
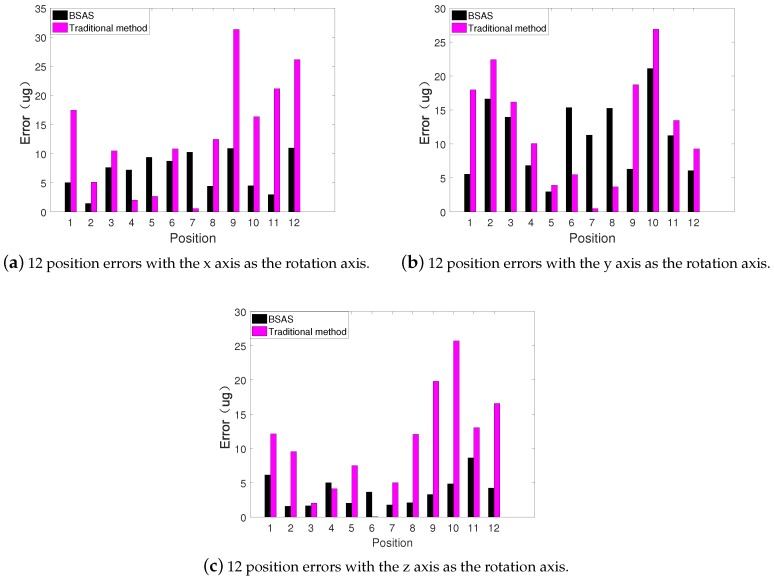
36 position error.

Seen from the Figure 9, the 36 position measurement error range of the traditional calibration scheme is in 1 ug ∼ 35 ug, while the measurement error based on the proposed scheme is varied from 1 ug to 25 ug. In addition, Table 5 records the mean and standard deviation of measurement error. The average measurement error of the traditional calibration scheme is 12.025 ug, while that of the proposed scheme is only 7.253 ug. In terms of standard deviation, the traditional scheme is 8.296 ug, and the proposed scheme is only 4.786 ug.

In summary, the experiment of 36 position triaxial acceleration measurement demonstrate that no matter whether it is accuracy or stability, the performance of the proposed method is much better than the traditional method.

### 5.2. Static Navigation Experiment Based on SINS

Some studies have shown that the accuracy of static inertial navigation is greatly affected by the calibration error of IMU [37,38]. Therefore, in this section, the performance of the proposed scheme will be further verified through the static navigation experiment based on SINS.

To begin with, the IMU is placed on the three-axis turntable and keep it at a fixed angle. After orientation alignment, IMU is used to collect raw data, and then the positioning error is calculated with different calibration parameters (traditional method and proposed method) by SINS. This process is done in the navigation processor.

**Figure 10:**
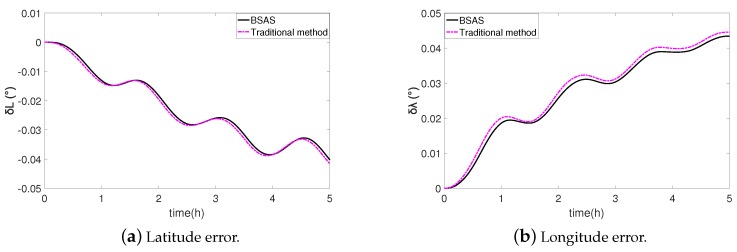
Error of latitude and longitude.

Figure 10 is the longitude and latitude error curve of 5 hour static navigation with two calibration methods. Firstly, the accelerometer is calibrated by the traditional method, and the latitude error is $-0.0416^{\circ }$ and the longitude error is $0.0445^{\circ }$. Afterwards, the BSAS algorithm is used to perform in-field recalibration, and the latitude error and longitude error are $-0.0402^{\circ }$ and $0.0434^{\circ }$ respectively, which are better than the results of traditional methods. Moreover, the positioning error can be calculated as Equation (23), where *R* is the radius of earth, δL(t) is the latitude error at time *t*, and δλ(t) is the longitude error at time *t*. The results are shown in Figure 10.

(23) $$P_{e}\left(t\right)=R\sqrt{\delta L^{2}\left(t\right)+\delta \lambda^{2}\left(t\right)\cos^{2}\left(L\left(t\right)\right)}$$

**Figure 11:**
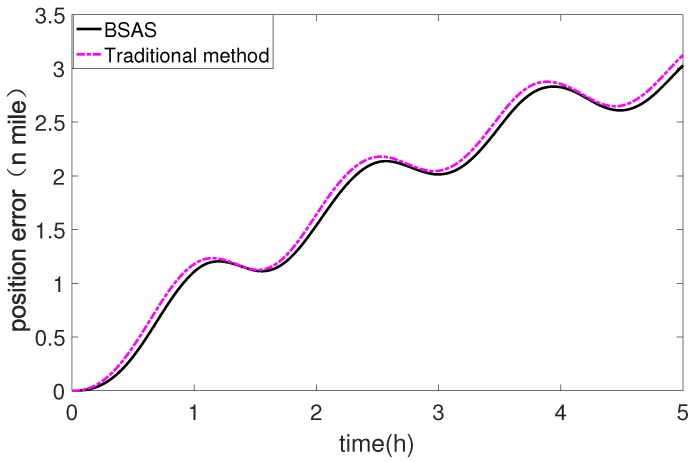
Positioning error of static navigation.

It can be seen from Figure 11, the proposed in-field recalibration method represented by black curve has a satisfactory calibration accuracy, and the positioning error has been smaller than the traditional calibration method in the 5 hours static navigation experiment. More error information about the two methods is shown in Table 6.

In Table 6, after recalibration by using the proposed scheme, the maximum error of 5 h static navigation is 3.0273 nautical miles, and the mean value of error is 1.7583 nautical miles. For the traditional scheme, the maximum error is 3.1229 nautical miles and the mean value of error is 1.8165 nautical miles. The positioning error results clearly show that the proposed scheme has higher calibration accuracy than the traditional scheme.

## 6. Conclusions

This paper presents a new in-field calibration method to recalibrate the triaxial accelerometer. This method is based on a new meta-heuristic intelligent optimization algorithm, beetle swarm antenna search algorithm. First of all, in order to ensure higher calibration accuracy, a nonlinear mathematical model of triaxial accelerometer with 15 calibration parameters is established. What’s more, by analyzing the sensitivity of each parameter to the local gravity value, the optimal measurement location scheme is designed in this paper. Finally, according to the characteristics of the BSAS algorithm, the 15 calibration parameters are classified first and then recalibrated in order. In order to verify the performance of proposed method, simulations and experiments are performed separately.

Simulation results show that the BSAS algorithm is significantly superior to the basic BAS algorithm in terms of calibration speed and accuracy. Furthermore, the robustness and stability of the BSAS algorithm is also better than the basic BAS algorithm. Therefore, the BSAS algorithm is more suitable for solving complex problems like the in-field calibration. Then, the effectiveness of the BSAS algorithm is proved by 24 position measurement experiment and static navigation experiment. By comparing with the traditional calibration method, it shows that the results of in-field recalibration by BSAS algorithm can meet the requirements of high precision navigation. In addition, the proposed scheme has the advantages of simple design, fast calibration speed, and does not require any external equipment, so it can be applied to engineering as an alternative to the traditional scheme.

However, the calibration technique based on the BSAS algorithm is still in the exploratory stage. In the future, we will continue to focus on the development and improvement of new algorithms, and achieve online applications for the calibration of inertial sensors.

## Figures and Tables

**Table 1:** Real value setting of calibration parameter.

Parameter	Real Values
$K_{ax}$, $K_{ay}$, $K_{az}$ (plus/(m/s${}^{2}$))	−734.949141, −738.738914, −714.409875
$b_{x0}$, $b_{y0}$, $b_{z0}$ (m/s${}^{2}$)	0.00654668, −0.04285332, 0.01471738
$S_{axz}$, $S_{axy}$, $S_{ayz}$	0.00050230, 0.00068317, −0.00048500
$S_{ayx}$, $S_{azy}$, $S_{azx}$ (deg)	−0.00035363, 0.00023193, −0.00003365
$K_{2ax}$, $K_{2ay}$, $K_{2az}$ (plus/(m/s${}^{2}$)${}^{2}$)	0.003654, 0.000644, −0.002654

**Table 2:** Parameters setting of BSAS algorithm.

**Beetles** **Numbers (*m*)**	**Position Update** **Probability Constant ($p_{\mathrm{pos}}$)**	**Step Size Update** **Probability Constant ($p_{\mathrm{st}}$)**	**Maximum Number of** **Invalid Searches ($n_{\mathrm{st}}$)**
10	0.8	0.8	2
**Searching** **Step Size (s)**	**Attenuation Coefficient** **of Step Size ($c_{s}$)**	**Searching** **Distance (d)**	**Attenuation Coefficient** **of Searching Distance ($c_{d}$)**
0.98	0.9	2	0.9

**Table 3:** 50 simulation results of 15 calibration parameters.

		BSAS			Basic BAS		
Paramters	Real Values	Mean	RMSE	SD	Mean	RMSE	SD
$K_{ax}$ (plus/(m/s${}^{2}$))	−734.949141	−734.949176	$3.535\times 10^{-5}$	$4.717\times 10^{-9}$	−737.770451	2.9442	0.8418
$K_{ay}$ (plus/(m/s${}^{2}$)	−738.738914	−738.738963	$4.945\times 10^{-5}$	$8.574\times 10^{-9}$	−735.325275	3.4575	0.5493
$K_{az}$ (plus/(m/s${}^{2}$)	−714.409875	−714.409849	$2.523\times 10^{-5}$	$1.709\times 10^{-7}$	−716.269090	2.0935	0.9624
$b_{x0}$ (m/s${}^{2}$)	0.00654667	0.00654670	$3.155\times 10^{-8}$	$2.329\times 10^{-8}$	0.00670143	0.002	0.002
$b_{y0}$ (m/s${}^{2}$)	−0.04285331	−0.04285266	$6.482\times 10^{-7}$	$2.013\times 10^{-8}$	−0.04287217	0.00012	0.00012
$b_{z0}$ (m/s${}^{2}$)	0.01471738	0.01471718	$2.039\times 10^{-7}$	$1.550\times 10^{-8}$	0.01479223	0.0021	0.0021
$S_{axz}$ (deg)	0.00050230	0.00050739	$6.973\times 10^{-6}$	$4.752\times 10^{-6}$	-	-	-
$S_{axy}$ (deg)	0.00068317	0.00064748	$4.350\times 10^{-5}$	$2.488\times 10^{-5}$	-	-	-
$S_{ayz}$ (deg)	−0.00048500	−0.00049006	$6.945\times 10^{-6}$	$4.751\times 10^{-6}$	-	-	-
$S_{ayx}$ (deg)	−0.00035363	−0.00035271	$3.499\times 10^{-6}$	$3.378\times 10^{-6}$	−0.00030826	$5.285\times 10^{-5}$	$2.032\times 10^{-5}$
$S_{azy}$ (deg)	0.00023193	0.00026761	$4.350\times 10^{-5}$	$2.488\times 10^{-5}$	-	-	-
$S_{azx}$ (deg)	−0.00003365	−0.00003447	$3.442\times 10^{-6}$	$3.343\times 10^{-6}$	-	-	-
$K_{2ax}$ plus/(m/s${}^{2}{)}^{2}$)	0.003654	0.003653	$1.107\times 10^{-6}$	$5.578\times 10^{-9}$	−0.013728	0.0607	0.0582
$K_{2ay}$ plus/(m/s${}^{2}{)}^{2}$)	0.000644	0.000643	$6.373\times 10^{-7}$	$6.384\times 10^{-8}$	0.000734	0.0016	0.0017
$K_{2az}$ plus/(m/s${}^{2}{)}^{2}$)	−0.002654	−0.002655	$8.715\times 10^{-7}$	$7.109\times 10^{-9}$	−0.040289	0.0614	0.0485

**Table 4:** Parameters of the gyroscope and accelerometer.

Parameter Item	Parameter Values
Accelerometer constant bias (μg)	10
Accelerometer dynamic range (g)	±60
Gyro constant bias (${}^{\circ }$/h)	0.01

**Table 5:** 36 position error results.

Method	Mean Value of Error (ug)	Standard Deviation of Error (ug)
Traditional method	12.025	8.296
Proposed method	7.253	4.786

**Table 6:** Static navigation results.

Method	Maximum Error (nmile)	Mean Value of Error (nmile)
Traditional method	3.1229	1.8165
Proposed method	3.0273	1.7583

## References

[1] Xiong H., Tang J., Xu H., Zhang W., Du Z. (2017). A robust single GPS navigation and positioning algorithm based on strong tracking filtering. IEEE Sens. J..

[2] Liu J., Pu J., Sun L., He Z. (2019). An Approach to Robust INS/UWB Integrated Positioning for Autonomous Indoor Mobile Robots. Sensors.

[3] Gross J.N., Gu Y., Rhudy M.B. (2015). Robust UAV relative navigation with DGPS, INS, and peer-to-peer radio ranging. IEEE Trans. Autom. Sci. Eng..

[4] Zhang T., Chen L., Li Y. (2016). AUV underwater positioning algorithm based on interactive assistance of SINS and LBL. Sensors.

[5] Titterton D., Weston J.L., Weston J. (2004). Strapdown Inertial Navigation Technology.

[6] Fontanella R., Accardo D., Moriello R.S.L., Angrisani L., De Simone D. (2018). MEMS gyros temperature calibration through artificial neural networks. Sens. Actuators Al.

[7] Cao H., Zhang Y., Shen C., Liu Y., Wang X. (2018). Temperature energy influence compensation for MEMS vibration gyroscope based on RBF NN-GA-KF method. Shock. Vib..

[8] Poddar S., Kumar V., Kumar A. (2017). A comprehensive overview of inertial sensor calibration techniques. J. Dyn. Syst. Meas. Contr..

[9] Bonnet S., Bassompierre C., Godin C., Lesecq S., Barraud A. (2009). Calibration methods for inertial and magnetic sensors. Sens. Actuators A.

[10] Ferraris F., Grimaldi U., Parvis M. (1995). Procedure for effortless in-field calibration of three-axis rate gyros and accelerometers. Sens. Mater..

[11] Shin E.H., El-Sheimy N. (2002). A new calibration method for strapdown inertial navigation systems. ZFV.

[12] Syed Z.F., Aggarwal P., Goodall C., Niu X., El-Sheimy N. (2007). A new multi-position calibration method for MEMS inertial navigation systems. Meas. Sci. Technol..

[13] Won S.h.P., Golnaraghi F. (2009). A triaxial accelerometer calibration method using a mathematical model. IEEE Trans. Instrum. Meas..

[14] Cai Q., Song N., Yang G., Liu Y. (2013). Accelerometer calibration with nonlinear scale factor based on multi-position observation. Meas. Sci. Technol..

[15] Ye L., Guo Y., Su S.W. (2017). An efficient autocalibration method for triaxial accelerometer. IEEE Trans. Instrum. Meas..

[16] Wang Z., Cheng X., Fu J. (2019). Optimized Multi-Position Calibration Method with Nonlinear Scale Factor for Inertial Measurement Units. Sensors.

[17] Chen D., Li S. (2019). New super-twisting zeroing neural-dynamics model for tracking control of parallel robots: A finite-time and robust solution. IEEE Trans. Cybern..

[18] Chen D., Li S. (2019). New disturbance rejection constraint for redundant robot manipulators: An optimization perspective. IEEE Trans. Ind. Inf..

[19] Jiang X., Li S. (2017). BAS: beetle antennae search algorithm for optimization problems. arXiv.

[20] Zhang Y., Li S., Xu B. (2019). Convergence analysis of beetle antennae search algorithm and its applications. arXiv.

[21] Wu Q., Shen X., Jin Y., Chen Z., Li S., Khan A.H., Chen D. (2019). Intelligent beetle antennae search for UAV sensing and avoidance of obstacles. Sensors.

[22] Fei S.W., He C.X. (2019). Prediction of dissolved gases content in power transformer oil using BASA-based mixed kernel RVR model. Int. J. Green Energy.

[23] Wu Q., Ma Z., Xu G., Li S., Chen D. (2019). A Novel Neural Network Classifier Using Beetle Antennae Search Algorithm for Pattern Classification. IEEE Access.

[24] Fan Y., Shao J., Sun G. (2019). Optimized PID Controller Based on Beetle Antennae Search Algorithm for Electro-Hydraulic Position Servo Control System. Sensors.

[25] Li Q., Wang Z., Wei A. (2019). Research on Optimal Scheduling of Wind-PV-Hydro-Storage Power Complementary System Based on BAS Algorithm. MSE.

[26] Chen C., Tello Ruiz M., Lataire E., Delefortrie G., Mansuy M., Mei T., Vantorre M. Ship manoeuvring model parameter identification using intelligent machine learning method and the beetle antennae search algorithm. Proceedings of the ASME 2019 38th International Conference on Ocean, Offshore and Arctic Engineering.

[27] Sun Y., Zhang J., Li G., Wang Y., Sun J., Jiang C. (2019). Optimized neural network using beetle antennae search for predicting the unconfined compressive strength of jet grouting coalcretes. Int. J. Numer. Anal. Methods Geomech..

[28] Wang J., Chen H., Yuan Y., Huang Y. (2019). A novel efficient optimization algorithm for parameter estimation of building thermal dynamic models. Build. Environ..

[29] Wang J., Chen H. (2018). BSAS: Beetle Swarm Antennae Search Algorithm for Optimization Problems. arXiv.

[30] Xie S., Garofano V., Chu X., Negenborn R.R. (2019). Model predictive ship collision avoidance based on Q-learning beetle swarm antenna search and neural networks. Ocean Eng..

[31] Lin X., Liu Y., Wang Y. Design and Research of DC Motor Speed Control System Based on Improved BAS. Proceedings of the 2018 Chinese Automation Congress (CAC).

[32] Rong T., Shen C., Yuan Z. (2000). The Principle of Measuring the Displacement with Accelerometer and the Error Analysis. J. Huazhong Univ. Sci. Techno..

[33] Särkkä O., Nieminen T., Suuriniemi S., Kettunen L. (2017). A multi-position calibration method for consumer-grade accelerometers, gyroscopes, and magnetometers to field conditions. IEEE Sens. J..

[34] Yang J., Wu W., Wu Y., Lian J. (2012). Thermal calibration for the accelerometer triad based on the sequential multiposition observation. IEEE Trans. Instrum. Meas..

[35] (2014). A multi-position self-calibration method for dual-axis rotational inertial navigation system. Sens. Actuators Al.

[36] Hussain K., Salleh M.N.M., Cheng S., Shi Y. (2019). Metaheuristic research: A comprehensive survey. Artif. Intell. Rev..

[37] Tan C.W., Park S. (2005). Design of accelerometer-based inertial navigation systems. IEEE Trans. Instrum. Meas..

[38] El-Diasty M., Pagiatakis S. (2008). Calibration and stochastic modelling of inertial navigation sensor errors. JGPS.

